# Systematic review of efficacy of hair transplantation in patients with classic lichen planopilaris and frontal fibrosing alopecia: revisiting the current evidence

**DOI:** 10.1016/j.abd.2026.501426

**Published:** 2026-08-12

**Authors:** Rodolfo David Palacios-Diaz, Miguel Antonio Lasheras-Pérez

**Affiliations:** a Dermatology Department, Hospital Comarcal de Vinaròs, Castellón, Spain; b Dermatology Department, Hospital Universitari i Politècnic La Fe, Valencia, Spain

**Keywords:** Frontal fibrosing alopecia, Hair transplantation, Lichen planopilaris

## Abstract

**Background:**

Lichen planopilaris (LPP) is the most common form of primary cicatricial alopecia. Areas of alopecia are permanent; thus, hair transplantation becomes a potential cosmetic alternative.

**Objective:**

To review the evidence of the efficacy of hair transplantation in patients with LPP.

**Methods:**

The authors conducted a systematic review across four databases: Web of Science, PubMed, SciELO, and Cochrane. The authors analyzed studies describing the use of hair transplantation in patients with LPP, in any of its subtypes. Articles published from the inception of each database through October 2025 were included. Data from each study were summarized in tables. When possible, quantitative variables were grouped and expressed as frequencies or means.

**Results:**

The authors included thirteen studies (113 patients): eight were case series (61.5%) and five articles were individual case reports (38.5%). 64.8% patients had frontal fibrosing alopecia, while 35.2% had the classic subtype. Hair transplantation was performed in patients with no clinically active disease in ten studies (101 patients). Nevertheless, the duration of quiescence prior to transplantation varied widely, ranging from 0 to 60 months. In most cases, survival rates were between from 80%‒100% at 6‒12 months, 71%‒100% up to 24-months, and below 41% after the second year.

**Study limitations:**

No prospective studies or clinical trials were identified. There was a marked heterogeneity in the reported data, and follow-up times were inconsistent.

**Conclusions:**

Hair transplantation may be considered as a cosmetic alternative for patients with LPP. However, a clear decline in graft survival rate was noted after the second year.

## Introduction

Alopecia is defined as the partial or complete loss of hair in areas where hair is normally expected to grow.^1^ Hair loss has a significant impact on psychosocial function and can provoke anxiety and distress that may be disproportionate to its apparent objective severity.^2^, ^3^

Lichen Planopilaris (LPP) is the most common primary scarring alopecias.^4^, ^5^ Three clinical subtypes have been described: the classic subtype, Frontal Fibrosing Alopecia (FFA) and the Graham-Little-Piccardi-Lassueur syndrome.^6^, ^7^, ^8^ The etiopathogenesis of LPP is not fully understood. However, it is believed that an immune privilege collapse, induced by interferon-gamma, occurs in the follicular bulge of patients with LPP.^9^ This phenomenon allows for differentiation between lesional and non-lesional scalp in affected individuals.^9^ Due to the permanent nature of the condition, early management is essential.

Multiple medical therapies have been described, with varying degrees of efficacy. The main goals of treatment are to alleviate symptoms, reduce disease progression, and improve hair density.^10^, ^11^, ^12^ Surgical hair restoration has emerged as a cosmetic alternative. Surgical approaches include excision of alopecic scalp areas, scalp flaps, and hair transplantation.^13^

Hair transplantation aims to cover areas of scarring alopecia. The procedure involves relocating hair follicles from unaffected areas to recipient sites, with the expectation that transplanted follicles will continue producing healthy terminal hairs.^14^ Techniques for harvesting donor follicles include strip harvesting (follicular unit transplantation – FUT) and follicular unit extraction (FUE).^15^ The choice of technique typically depends on the clinical characteristics of the alopecia, the patient’s specific needs, and the surgical expertise of the treating team.

Hair transplantation in scarring alopecia presents unique challenges. Scarring alopecias alter the anatomy of the recipient site and may also affect potential donor areas. Other factors to consider include the potential for disease reactivation, donor site availability, scalp laxity, individual healing patterns, vascularization of both donor and recipient regions, and the location of the resulting scar.^16^

There are a few reports of patients with primary scarring alopecias undergoing hair transplantation. The use of this technique remains controversial due to the high likelihood of poor follicular growth post-transplant.^17^, ^18^ Furthermore, cases of LPP developing after hair transplantation have been reported.^18^ The most recent systematic reviews addressing the topic of the present study were published in 2019 and 2021.^19^, ^20^ However, the first one included other types of primary scarring alopecia,^19^ and neither review considered the four most recent studies reporting this procedure, which contributed a substantial number of new cases. Consequently, the objective of this study is to review the current evidence regarding the efficacy of hair transplantation in patients with LPP.

## Material and methods

### Study design and eligibility criteria

A systematic review was conducted in accordance with the Preferred Reporting Items for Systematic Reviews and Meta-Analyses (PRISMA) methodology.^21^ The authors included case reports, case series, and randomized clinical trials published in English or Spanish that described the use of hair transplantation in patients with LPP, in any of its subtypes: classic, FFA, or Graham-Little-Piccardi-Lasseur syndrome. The authors excluded studies involving non-human subjects, secondary studies (narrative or systematic reviews), studies in which the type of scarring alopecia was not specified, studies that reported the use of artificial hair, and studies that did not report post-transplantation outcomes.

### Literature search

A comprehensive literature search was conducted across four databases: Web of Science, PubMed, SciELO, and Cochrane. Articles published from the inception of each database through October 2025 were included. Keywords used in the search included: “lichen planopilaris”, “frontal fibrosing alopecia”, “scarring alopecia”, “hair transplant”, and related terms, in both English and Spanish. The search strategy used in PubMed was as follows: ((lichen planopilaris) OR (“frontal fibrosing alopecia”) OR (“graham little piccardi lassueur”)) AND ((“hair transplantation”) OR (transplantation)). Search strategies for the remaining databases are provided in Supplementary Table 1. Additionally, a manual search was performed to identify studies referenced in the initially retrieved reports. Abstracts were screened by two independent reviewers (RDPD and MALP) to identify all articles meeting the inclusion criteria. When discrepancies arose, the abstracts were re-examined jointly by both reviewers until a consensus was reached.

### Quality assessment

Study quality was assessed according to the levels of evidence established by the Oxford Centre for Evidence-Based Medicine.^22^ The 2011 Oxford Centre for Evidence-Based Medicine (OCEBM) Levels of Evidence is a ranking scheme that covers a range of clinical questions.^23^ The lower the risk of confounding (bias), the further to the left the type of evidence will lie.^23^ The systematic search did not identify any controlled clinical trials or cohort studies describing hair transplantation in patients with LPP.

### Data extraction and synthesis

The extracted data included patient demographics, clinical features of LPP, such as location, extent, clinical activity, prior and current treatments, and transplantation details such as the donor harvesting technique, number of follicular units transplanted, and outcome. Graft growth or graft survival can be evaluated by the persistence of follicular units or hair shafts. Since follicular units may contain varying numbers of hairs, assessing survival based on the number of hairs may provide more informative results than using follicular unit persistence alone.^24^ Given the absence of a standardized method, available data on either of these parameters ‒ or other metrics reported by the original authors, such as hair density (hairs/cm^2^), subjective assessments, among others ‒ were collected. For each study, data were extracted independently by two reviewers. When discrepancies arose, full-text manuscripts were re-examined jointly by both reviewers until consensus was reached. Data from each report were summarized in tables. When possible, quantitative variables were grouped and expressed as frequencies or means. In such cases, statistical analysis was performed using Microsoft Excel.

## Results

### Study characteristics

**Figure 1:**
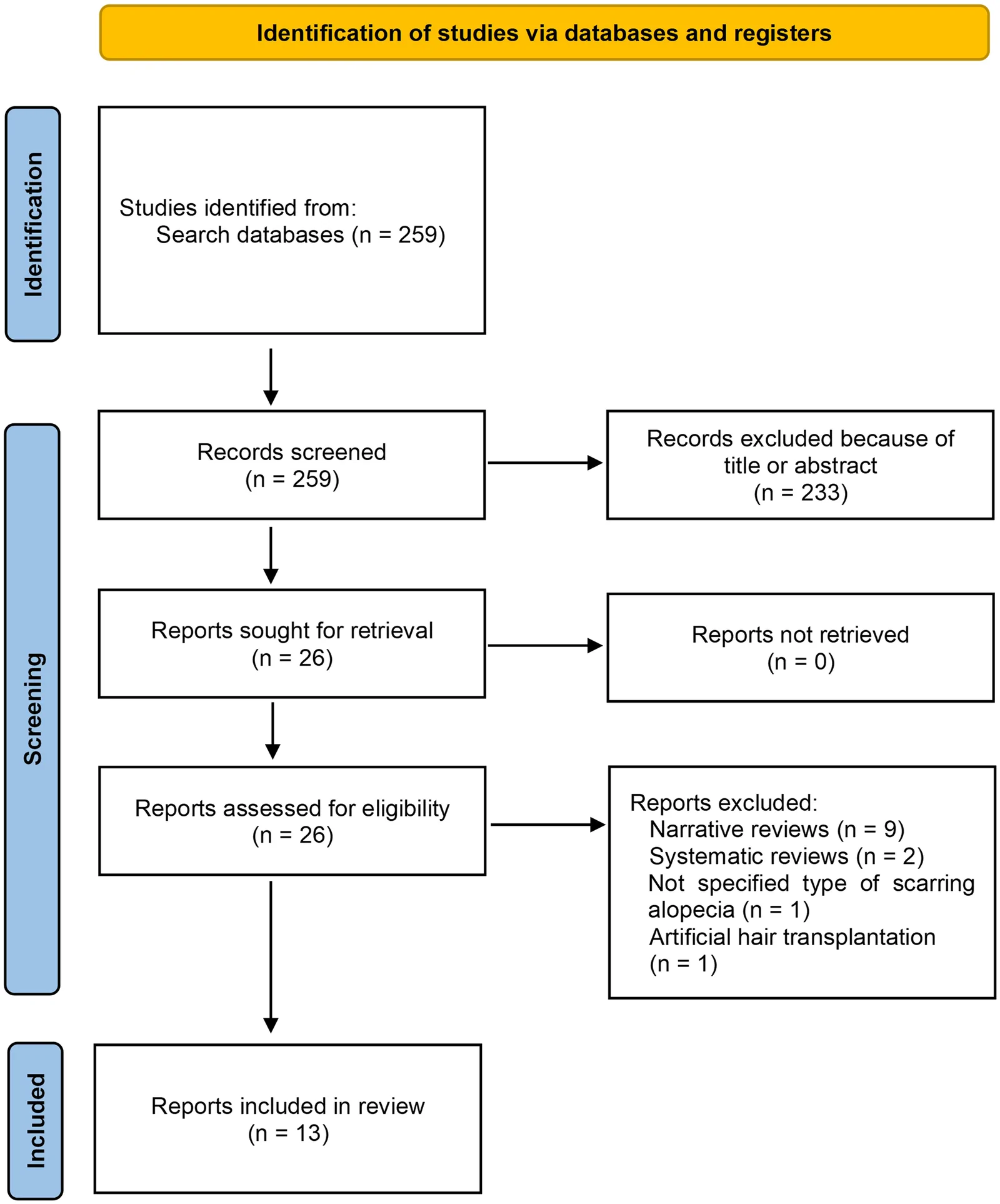
PRISMA flow diagram of the study selection process.

The selection process is illustrated in Fig. 1. After full-text review, thirteen studies were included. Of these, eight were case series (8/13; 61.5%) and five were individual case reports (5/13; 38.5%). Given that the studies are case reports or case-series, they were classified as level 4 evidence according to the Oxford Centre for Evidence-Based Medicine (Supplementary Table 2).

### Patient characteristics

The studies included a total of 113 patients (Table 1).^25^, ^26^, ^27^, ^28^, ^29^, ^30^, ^31^, ^32^, ^33^, ^34^, ^35^, ^36^, ^37^ Two studies did not report the age or sex of the patients (n = 5). Among the remaining patients, 20.4% (22/108) were male and 79.6% (86/108) were female. The mean age was 48.9-years. In 105 patients, the subtype of LPP was reported or suggested. Of these, 64.8% (68/105) had frontal FFA, and 35.2% (37/105) had the classic subtype. The affected area was located exclusively in the scalp in eleven studies, the eyebrows in one study, and in both in one study.

**Table 1:** Summary of hair transplantation studies in patients with lichen planopilaris. Clinical and epidemiological characteristics of the transplanted patients.

Reference	Year	Type of study	N° patients / Sex	Mean age (y)	Clinical subtype^a^	Location	Activity (When last?)	Previous treatment for LPP
Podda M. et al.^25^	2000	Case series	2	NS	Classic	Scalp	Not (> 3y)	NS
Cevasco NC. et al.^26^	2007	Case series	3	NS	NS	Scalp	Not (NS time)	Yes
Nusbaum BP. et al.^27^	2010	Case report	1 / M	44	FFA	Scalp	Not (10 m)	Yes
Gurfinkiel A. et al.^28^	2011	Case report	1 / W	62	FFA	Scalp	Yes	Yes
Jiménez F. et al.^29^	2013	Case series	3 / W	70	FFA	Scalp	Not (NS time)	Yes
Greco CF. et al.^30^	2015	Case report	1 / W	54	Classic	Scalp	Not (3y)	Yes
Saxena K. et al.^31^	2016	Case report	1 / M	24	Classic	Scalp	Not (2y)	Yes
Liu YS. et al.^32^	2018	Case series	2 / W	52.5	FFA (1) / Classic (1)	Scalp	Not (2.5y)	Yes
Scribel M. et al.^33^	2018	Case report	1 / W	57	FFA	Scalp	NS	NS
Vañó-Galván S. et al.^34^	2019	Case series	(n = 51), 48 W, 3M	54	FFA	Scalp & eyebrows	Not (15 m^‡^)	NS
Audickaite A. et al.^35^	2020	Case series	(n = 10), 9 W, 1M	47.5	FFA	Eyebrows	NS	Yes (9/10)
Daruwalla SB. et al.^36^	2021	Case series	(n = 32), 18 W, 14M	31.5	Classic	Scalp	Not (LPPAI = 0) (> 6 m)	NS
Osipowicz K. et al.^37^	2025	Case series	(n = 5), 4 W, 1M	50.6	NS	Scalp	Not (5–40 m)	NS

a When the clinical subtype of LPP was not described, it was considered as not specified (NS); Although authors reported a mean time of disease stabilization of 15-months, range was from 0- to 60-months. W, Woman; M, Man; NS, Not Specified; LPP, Lichen Planopilaris; FFA, Frontal Fibrosing Alopecia; LPPAI, *Lichen Planopilaris Activity Index*; m, months; y, years.

Hair transplantation was performed in patients with no clinically active disease in ten studies (101-patients). The minimum period of clinically stable disease prior to transplantation varied. Reported inactive periods ranged from zero months to more than three years. Notably, in the study by Vañó-Galván S et al., although the mean duration of clinical stability was 15-months, the reported range was from 0- to 60-months.^34^ In most reports, the patients had received specific treatment for LPP before undergoing transplantation. In one study, transplantation was performed in a patient with signs of active disease, and in two studies (11-patients), disease activity was not specified.

### Hair transplant characteristics

Hair transplant features and post-transplant outcomes are summarized in Table 2. The follicular unit harvesting method was reported in twelve out of thirteen studies (92.3%). In one article, the authors did not specify whether follicular units were obtained via 5 mm punch biopsies or strip harvesting. In the remaining eleven articles, which included 108-patients, the extraction method was clearly stated. FUE was used in 53.7% of patients (58/103), while FUT was used in 46.3% (50/108). All patients (50/50) who underwent FUT had the FFA subtype. In contrast, among those treated with FUE, 60.4% (35/58) had the classic phenotype, 31% (18/58) had FFA, and in 8.6% (5/58) the clinical subtype was not specified.

**Table 2:** Summary of hair transplantation studies in patients with lichen planopilaris. Transplant characteristics and outcomes.

Reference	Transplant type	LPP treatment during transplant	Transplant test	Follicular units	LPP treatment after transplant	Outcome
Podda M. et al.^25^	NS‡	NSᶜ	Yes	NS	NS	- “*Long term*”: > 95%ᵇ.
						- Two to five transplant sessions were performed every 3 to 6 m.
Cevasco NC. et al.^26^	NS	NSᶜ	NS	NS	NS	- Authors evaluated hair regrowth in the active perimeter: one patient as “worse”, one as “fair”, one as “good”.
						- Not specified time of follow-up.
Nusbaum BP. et al.^27^	FUT	Yes	Yes†	82	Yes*	- 15 m: “excellent” (no hair counts were performed) // 4y: 0%
						*After 15 m, the patient suspended active treatments
Gurfinkiel A. et al.^28^	FUE	Yes	Not	NS (2000 hairs)	Yes	- 6y: 85%^a^
Jiménez F. et al.^29^	FUE	Yes	Yes†	50	NS	- 12–18 m: 80%–100%ᵇ
						- After 2.5y: ≤40%ᵇ
Greco CF. et al.^30^	FUE	Yes	Yes	2606	Not	- “Good outcome” at 6 m.
Saxena K. et al.^31^	FUE	Not	Yes	900	Not	- 10 m: 80%ᵇ
Liu YS. et al.^32^	FUE	Not	Not	455	Not	- Patient 1: 56 hairs/cm^2^ (frontal hairline)
						- Patient 2: 58 hairs/cm^2^ (frontal hairline) y 126 hairs/cm^2^ (vertex)
						Stable after a follow-up period of 4y and 3y – 4 m, respectively.
Scribel M. et al.^33^	FUE	Yes	Not	NS	Yes*	- 24 m: “Successful”
						*LPP treatment was initiated when disease activity was observed.
Vañó-Galván S. et al.^34^	FUE (7) / FUT (44)	Yes	Not	1345	Yes	- 12 m: 87% // 24 m: 71% // 36 m: 60% // 60 m: 41%
						12 patients with follow-up ≥ 5y had a graft survival rate < 60%
Audickaite A. et al.^35^	FUE (5) / FUT (5)	NS	Not	356α	NS	- 6 – 12 m: 8/10: 80–100% a
						2/10: 50–60% a
						- 3–4 a: 1/4: “Good result” (⁓80%)^a^
						3/4: “Poor result”
						- 4 patients had a second hair transplant
Daruwalla SB. et al.^36^	FUE	Not	Yes†	100	Not	- 3 m: 37.8%^a^ // 6 m: 52.44%^a^ // 12 m: 78.62%^a^ // 24 m: 79.96% a
Osipowicz K. et al.^37^	FUE	Not	Not	2232γ	Yes*	- Authors assessed satisfaction by a 5-point Likert scale.
						6 months: 2/5 (40%) strong dissatisfaction, 2/5 (40%) satisfied, 1/5 (20%) very satisfied.
						12 months: 5/5 (100%) very satisfied
						Two patients had multiple surgeries to achieve a positive outcome.

‡ Hair grafts were obtained with 5 mm punch biopsy or strips.

† Data from transplantation test is presented in the table because a later hair transplant was not performed.

a Hair survival rate.

ᵇ Graft survival rate.

ᶜ Although it was not specified whether the patients were on LPP treatment, the authors mentioned that LPP were inactive or end-stage.

α Mean of transplanted follicular units in both eyebrows for each patient.

γ Some patients had multiple transplantations; mean of number of grafts were considered for each individual procedure. m, months; y, years; FUE, Follicular Unit Extraction; FUT, Follicular Unit Transplantation; NS, Not Specified.

The authors mentioned performing a transplant test in six studies. However, in three of these, no subsequent full transplantation was performed. In six studies, patients were receiving active treatment for LPP at the time of transplantation. Two studies did not specify whether patients were under active treatment, but noted that LPP was in an inactive or end-phase. In one study, per protocol, LPP medical therapies had been discontinued for 6- to 12-months before transplantation.^36^

The number of transplanted Follicular Units (FU) varied. In reports where only test transplant results were reported, the number of FU ranged from 50 to 100. In the study reporting eyebrow-specific data, the average number of transplanted FU for both eyebrows was 356. Four studies did not report the number of FU. In the remaining five reports, the number ranged widely from 455 to 2606 FU. In five studies, patients received active treatment for LPP after the transplantation. In another four, no post-transplant active treatment was indicated. The post-transplant therapeutic approach was not specified in the remaining four studies.

### Graft survival and outcome

The studies reported varying follow-up durations and methods for assessing graft survival (Table 2).^25^, ^26^, ^27^, ^28^, ^29^, ^30^, ^31^, ^32^, ^33^, ^34^, ^35^, ^36^, ^37^ Among the studies with follow-up periods between six and twelve months (four studies; 94-patients), favorable hair growth was observed in the majority of cases (92/94; 97.9%), with reported graft survival rates ranging from 80% to 100%. In two patients, hair survival rates between 50% and 60% were noted. One case report described the outcome at six months as “good”, but no further follow-up was documented. In three studies (84-patients) with follow-up periods between twelve and twenty-four months, graft survival rates ranged from 71% to 100%. Additionally, in one article, hair growth at fifteen months post-transplant was described as “excellent”, and in another, the result was considered “successful” at twenty-four months. One study reported patient satisfaction as a hair transplantation outcome, showing variable results at six months but high satisfaction at twelve months.

In six studies (62-patients), graft survival rates were reported beyond twenty-four months after transplantation. Only two patients had graft survival rates between 80% and 85%. In contrast, the majority (55/62; 88.7%) had survival rates equal to or below 41%. In one case, a complete absence of hair regrowth was reported four years after an initially “excellent” result at fifteen months. Similarly, three patients, who had graft survival rates of 80%–100% at six and twelve months, showed “poor” hair growth three to four years later. In two patients, post-transplant evolution was assessed based on hair density per cm^2^. In both cases, the authors described hair growth as “stable” at four years of follow-up.

One study reported long-term graft survival greater than 95%, although the exact duration of follow-up was not specified. In another study, the specific timing of outcome assessment was not mentioned; however, the authors reported progression of LPP in one patient, disease instability with minimal hair growth in another, and disease stabilization with more-than-minimal growth in a third patient.

## Discussion

LPP is the most common type of scarring alopecia and has a significant impact on quality of life.^38^ Early diagnosis and prompt treatment are essential to halt disease activity. However, its management remains challenging, as responses to conventional therapies are often incomplete or absent.^6^ As a result, disease progression may lead to the development of scarring alopecic plaques.

Hair transplantation in LPP is controversial, mainly due to the risk of poor follicular growth and consequent graft failure. Additional concerns include determining the optimal timing of the procedure, the most suitable method for follicular unit extraction, and the potential need for continued active treatment for LPP following transplantation.

In this review, the authors found that graft survival was generally high (survival rate > 80%) during the first year in most patients. Between the first and second year, hair growth rates remained above 70%. A recent meta-analysis reported high graft survival rates in non-scarring alopecia (84.9%), with a mean follow-up of 1.2-years post-surgery, and in secondary scarring alopecia (88.6%), with a mean follow-up of 11.2-months, using only micrografts.^39^ Thus, in the short and medium term, graft survival in LPP appears comparable or only slightly lower than in both non-scarring and secondary scarring alopecia.

However, in the present review, after the second year, the survival rate declined to ≤ 41% in the majority of patients (89%), despite initially favorable growth. In one study, a biopsy of a residual follicular unit revealed histopathological findings consistent with FFA.^29^ This may suggest that the recipient site had ongoing subclinical inflammatory activity, despite apparent clinical stability, that LPP-related inflammation was predominant in the recipient area, or that the donor follicles may have been affected by subclinical LPP despite the absence of clinical or trichoscopic signs.^29^, ^35^

In the present review, ten studies reported clinical inactivity at the time of transplantation. LPP is considered an “unstable” scarring alopecia due to its tendency to progress and relapse intermittently over time.^40^ Defining stability in LPP ‒ whether in the classic subtype or FFA ‒ is inherently complex. The Lichen Planopilaris Activity Index has been proposed as a tool to assess disease activity and progression.^41^ However, it is not a standardized tool, and in fact, only one study in our review used it.^36^

Thus, no sensitive biomarker has yet been identified to determine disease remission.^35^ Consequently, the assessment of stability largely relies on the clinician’s judgment. Moreover, despite an apparently inactive state, the microtrauma induced during hair transplantation may reactivate the disease or even trigger *de novo* development of LPP.^21^, ^42^ Additionally, follicular inflammation has been reported in areas affected by FFA, although not in the transplanted follicles.^33^

The included studies reported variable durations of clinical stability prior to transplantation, ranging from zero to up to five years.^25^, ^34^ Notably, one study described a case in which hair transplantation was performed following the failure of conventional therapies in a patient with FFA.^28^ By contrast, the clinically recommended quiescent period prior to hair transplantation is typically one to two years without ongoing therapy.^40^, ^43^ However, there is no robust statistical evidence supporting this timeframe. The absence of individual patient data regarding clinical inactivity in LPP limits the ability to draw reproducible conclusions on its impact on hair graft survival rates.

The authors found a similar proportion of patients who had FUE and FUT techniques for follicular unit harvesting. However, all patients who underwent the strip technique presented with FFA. In contrast, the classic subtype was the most common presentation (70%) among those who underwent FUE. This may be related to the availability of donor follicle areas due to the different involvement patterns of LPP subtypes. Moreover, the possibility of performing FUE to harvest follicular units from the beard or anterior trunk allows for consideration of alternative donor sites.^31^, ^44^

In addition to the unstable course of LPP, the potential concomitant development of androgenetic alopecia (AGA) ‒ characterized by progressive hair thinning following a defined pattern ‒ must also be taken into account.^40^ This consideration is essential in transplantation planning in order to avoid “islands” of transplanted hair within a “sea” of hair affected by AGA.^40^ Hair transplantation does not halt the progression of AGA; therefore, long-term medical therapy to slow the process is essential.^45^

In parallel, maintenance medical treatment in patients with LPP after hair transplantation is not standardized. In our review, five studies explicitly reported the continuation of treatment following transplantation.^27^, ^28^, ^33^, ^34^^,^^37^ These studies described the use of 5-alpha reductase inhibitors,^27^, ^28^, ^37^ topical corticosteroids,^27^, ^28^ topical minoxidil,^28^, ^37^ and topical calcineurin inhibitors,^33^ with variable durations. Vañó-Galván S. et al. commented that patients received medical therapy for FFA after transplantation, but the authors did not specify the treatment regimens or their duration.^34^ Consequently, there is no significant evidence regarding the use of maintenance anti-inflammatory therapy, nor whether such treatment should be initiated only upon detection of clinical activity. Nonetheless, the use of 5-alpha reductase inhibitors may be considered as a strategy to slow AGA progression, while potentially stabilizing inflammation, particularly in patients with FFA.^46^, ^47^

A study proposed the use of a transplantation test with 400–500 follicular units in patients with AFF with active inflammation and fibrosis.^48^ The authors suggested that, after monitoring graft survival over a period of two to five years, a standard transplantation could be considered.^48^ In our review, six studies reported performing a transplantation test.^25^, ^27^, ^29^, ^30^, ^31^, ^36^ Although the reasons for not proceeding to subsequent transplantation were not always explicit, these may have included loss to follow-up with marked shedding of transplanted hair upon reassessment,^27^ follicle loss after 18-months to two years post-transplantation,^29^ or study design limitations.^36^

In three studies, after achieving an initially satisfactory outcome, hair transplantation was performed. Podda M et al. carried out a preliminary test with 50 follicular units.^25^ After four months, they performed between two and five additional sessions with 200 grafts, reporting acceptable graft survival.^25^ Greco C. et al. conducted a trial in 1 × 1 cm areas within the alopecic region.^30^ After confirming hair growth at four months, the authors proposed extending the transplantation to all affected alopecic areas.^30^ Similarly, Saxena K. et al. performed a test with 50-units and, after achieving an 80% growth rate at 10-months, completed the transplantation.^31^ Since graft survival progressively declines, particularly after the second year, defining the “success” of a transplantation trial is challenging without long-term follow-up.

This study had several limitations. First, despite the extensive search across multiple databases, there was a risk of missing potential articles indexed in alternative databases or published in languages other than English or Spanish. Moreover, the available studies on hair transplantation in patients with LPP were case reports or case series, and no prospective studies or clinical trials were identified. Even more, there was a marked heterogeneity among the included studies: some authors omitted relevant clinical information about patients and treatments, graft survival rates were not measured in a standardized manner, and follow-up times were inconsistent. Given that the primary goal of hair transplantation is cosmetic in nature, reporting bias cannot be excluded. Furthermore, because graft survival declines over time, long-term patient follow-up is essential. Future studies should therefore include larger cohorts, with homogeneous treatment protocols and follow-up regimens, to provide more robust conclusions.

## Conclusions

In this study, the authors investigated the application of hair transplantation in patients with LPP. The authors conducted a systematic review with the aim of identifying the clinical characteristics of patients and the efficacy of hair transplantation. The studies retrieved were case reports or case series. With the exception of one study, hair transplantation was performed in patients without clinical activity; however, the duration of inactivity varied widely, ranging from zero to 60-months.

The technique for follicular unit extraction varied, with a similar proportion of FUE and FUT, although the distribution of clinical phenotypes differed. A progressive decline in graft survival rate was observed. While survival rates were above 70% up to the second year, a clear decline was noted thereafter, with survival rates of 41% or lower.

Although hair transplantation may be considered a cosmetic option in patients with LPP, the data from the available studies are heterogeneous. Further research is needed to better define graft survival rates and to identify interventions that may improve outcomes. Prospective studies with larger patient cohorts and standardized treatment and follow-up protocols are required to generate more robust evidence.

## ORCID ID

Rodolfo David Palacios_Diaz: 0000-0002-8353-7481

Miguel Antonio Lasheras-Pérez: 0000-0001-7429-9592

## Research data availability

The entire dataset supporting the results of this study was published in this article.

## Financial support

None declared.

## Authors' contributions

Rodolfo David Palacios-Diaz: The study concept and design; data collection, or analysis and interpretation of data; statistical analysis; writing of the manuscript or critical review of important intellectual content; data collection, analysis and interpretation; approval of the final version of the manuscript.

Miguel Antonio Lasheras-Pérez: The study concept and design; data collection, or analysis and interpretation of data; effective participation in the research guidance; critical review of the literature; approval of the final version of the manuscript.

## Conflicts of interest

None declared.

## References

[1] Almudimeegh A., Alajlan A.H., Alrasheed A.I., Alrasheed M.I., Alqahtani A.K., Bin Idris R. (2025). The impact, prevalence, and association of different forms of hair loss among individuals with anxiety disorder: systematic review and meta-analysis. Medicine (Baltimore)..

[2] Collins K., Avram M.R. (2021). Hair transplantation and follicular unit extraction. Dermatol Clin..

[3] Hadshiew I.M., Foitzik K., Arck P.C., Paus R. (2004). Burden of hair loss: stress and the underestimated psychosocial impact of telogen effluvium and androgenetic alopecia. J Invest Dermatol..

[4] Vañó-Galván S., Saceda-Corralo D., Blume-Peytavi U., Cucchía J., Dlova N.C., Gavazzoni Dias M.F.R. (2019). Frequency of the types of alopecia at twenty-two specialist hair clinics: a multicenter study. Skin Appendage Disord..

[5] Griffin L.L., Michaelides C., Griffiths C.E.M., Paus R., Harries M.J. (2012). Primary cicatricial alopecias: a U.K. survey. Br J Dermatol..

[6] Svigos K., Yin L., Fried L., Lo Sicco K., Shapiro J. (2021). A practical approach to the diagnosis and management of classic lichen planopilaris. Am J Clin Dermatol..

[7] Vañó-Galván S., Molina-Ruiz A.M., Serrano-Falcón C., Arias-Santiago S., Rodrigues-Barata A.R., Garnacho-Saucedo G. (2014). Frontal fibrosing alopecia: a multicenter review of 355 patients. J Am Acad Dermatol..

[8] Alkhayal F.A., Alsudairy F., Al Mubarak L., Almohanna H.M. (2021). Graham-Little Piccardi Lassueur syndrome and review of the literature. Clin Case Rep..

[9] Harries M.J., Meyer K., Chaudhry I., Kloepper J.E., Poblet E., Griffiths C.E.M. (2013). Lichen planopilaris is characterized by immune privilege collapse of the hair follicle’s epithelial stem cell niche. J Pathol..

[10] Ezemma O., Devjani S., Kelley K.J., Senna M.M. (2023). Treatment modalities for lymphocytic and neutrophilic scarring alopecia. J Am Acad Dermatol..

[11] Vañó-Galván S., Trindade de Carvalho L., Saceda-Corralo D., Rodrigues-Barata R., Kerkemeyer K.L., Sinclair R.D. (2021). Oral minoxidil improves background hair thickness in lichen planopilaris. J Am Acad Dermatol..

[12] Bhoyrul B. (2024). The treatment of primary cicatricial alopecia is challenging. J Am Acad Dermatol..

[13] Liu Y.C.S., Jee S.H., Chan J.Y.L. (2018). Hair transplantation for the treatment of lichen planopilaris and frontal fibrosing alopecia: a report of two cases. Australas J Dermatol..

[14] Konior R.J., Simmons C. (2013). Patient selection, candidacy, and treatment planning for hair restoration surgery. Facial Plast Surg Clin North Am..

[15] Jimenez F., Vogel J.E., Avram M. (2021). CME article part II. hair transplantation: surgical technique. J Am Acad Dermatol..

[16] Kumar A.R., Ishii L.E. (2020). Hair transplantation for scarring alopecia. Facial Plast Surg Clin North Am..

[17] True R.H. (2021). Is every patient of hair loss a candidate for hair transplant? Deciding surgical candidacy in pattern hair loss. Indian J Plast Surg..

[18] Donovan J. (2012). Lichen planopilaris after hair transplantation: report of 17 cases. Dermatologic Surg..

[19] Ekelem C., Pham C., Atanaskova Mesinkovska N. (2019). A systematic review of the outcome of hair transplantation in primary scarring alopecia. Skin Appendage Disord..

[20] Lee J.A., Levy D.A., Patel K.G., Brennan E., Oyer S.L. (2021). Hair transplantation in frontal fibrosing alopecia and lichen planopilaris: a systematic review. Laryngoscope..

[21] Liberati A., Altman D.G., Tetzlaff J., Mulrow C., Gøtzsche P.C., Ioannidis J.P.A. (2009). The PRISMA statement for reporting systematic reviews and meta-analyses of studies that evaluate health care interventions: explanation and elaboration. J Clin Epidemiol..

[22] OCEBM Levels of Evidence Working Group. The oxford levels of evidence 2 [Internet]. Oxford: Oxford Centre for Evidence-Based Medicine. c2026 [cited 2026 Jan 6]. Available from: https://www.cebm.ox.ac.uk/resources/levels-of-evidence/ocebm-levels-of-evidence.

[23] Howick J, Chalmers I, Glasziou P, Greenhalgh T, Heneghan C, Liberati A, et al. Explanation of the 2011 Oxford Centre for Evidence-Based Medicine (OCEBM) levels of evidence. [Internet]. Oxford: Oxford Centre for Evidence-Based Medicine. c2026 [cited 2026 Jan 6]. Available from: https://www.cebm.ox.ac.uk/resources/levels-of-evidence/explanation-of-the-2011-ocebm-levels-of-evidence.

[24] Josephitis D., Shapiro R. (2018). FUT vs. FUE graft survival: a side-by-side study of 3 patients undergoing a routine 2,000+ graft hair transplantation. Int Soc Hair Restor Surg..

[25] Podda M., Spieth K., Kaufmann R. (2000). Er:YAG laser-assisted hair transplantation in cicatricial alopecia. Dermatologic Surg..

[26] Cevasco N.C., Bergfeld W.F., Remzi B.K., de Knott H.R. (2007). A case-series of 29 patients with lichen planopilaris: the Cleveland Clinic Foundation experience on evaluation, diagnosis, and treatment. J Am Acad Dermatol..

[27] Nusbaum B.P., Nusbaum A.G. (2010). Frontal fibrosing alopecia in a man: results of follicular unit test grafting. Dermatologic Surg..

[28] Gurfinkiel A., García Igarza H., Casas J., Kaminsky A. (2011). Trasplante capilar en una paciente con alopecia fibrosante frontal asociada con liquen escleroatrófico de vulva. Dermatol Argent..

[29] Jiménez F., Poblet E. (2013). Is hair transplantation indicated in frontal fibrosing alopecia? The results of test grafting in three patients. Dermatol Surg..

[30] Greco C.F., Chueco A., Acevedo A., Achenbach R.E., Dutto M. (2015). Trasplante de cabello en el liquen plano pilar. Rev Argent Dermatol..

[31] Saxena K., Saxena D.K., Savant S.S. (2016). Successful hair transplant outcome in cicatricial lichen planus of the scalp by combining scalp and beard hair along with platelet rich plasma. J Cutan Aesthet Surg..

[32] Liu Y.C.S., Jee S.H., Chan J.Y.L. (2018). Hair transplantation for the treatment of lichen planopilaris and frontal fibrosing alopecia: a report of two cases. Australas J Dermatol..

[33] Scribel M., Dutra H., Trüeb R. (2018). Autologous hair transplantation in frontal fibrosing alopecia. Int J Trichol..

[34] Vañó-Galván S., Villodres E., Pigem R., Navarro-Belmonte M.R., Asín-Llorca M., Meyer-González T. (2019). Hair transplant in frontal fibrosing alopecia: a multicenter review of 51 patients. J Am Acad Dermatol..

[35] Audickaite A., Alam M., Jimenez F. (2020). Eyebrow hair transplantation in frontal fibrosing alopecia: pitfalls of short- and long-term results. Dermatologic Surg..

[36] Daruwalla S.B., Dhurat R., Ghate S., Bhatt K. (2021). Long-term utility of follicular unit excision in lichen planopilaris - correlation of graft survival with histopathological and ultrasound biomicroscopic parameters. Dermatologic Surg..

[37] Osipowicz K., Turkowski P., Kowalewski C., Pach J., Regulski P., Wozniak K. (2025). Hair transplantation for lichen planopilaris: a case series of five patients. J Clin Med..

[38] Nasimi M., Ahangari N., Lajevardi V., Mahmoudi H., Ghodsi S.Z., Etesami I. (2020). Quality of life and mental health status in patients with lichen planopilaris based on Dermatology Life Quality Index and General Health Questionnaire-28 questionnaires. Int J Women Dermatol..

[39] Stoneburner J., Shauly O., Carey J., Patel K.M., Stevens W.G., Gould D.J. (2020). Contemporary management of alopecia: a systematic review and meta-analysis for surgeons. Aesthetic Plast Surg..

[40] Unger W., Unger R., Wesley C. (2008). The surgical treatment of cicatricial alopecia. Dermatol Ther..

[41] Chiang C., Sah D., Cho B.K., Ochoa B.E., Price V.H. (2010). Hydroxychloroquine and lichen planopilaris: efficacy and introduction of lichen planopilaris activity index scoring system. J Am Acad Dermatol..

[42] Jiménez F. (2012). Commentary: lichen planopilaris after hair transplantation. Dermatol Surg..

[43] Unger R., Shapiro R. (2023).

[44] Woods R., Campbell A.W. (2004). Chest hair micrografts display extended growth in scalp tissue: a case report. Br J Plast Surg..

[45] Jimenez F., Alam M., Vogel J.E., Avram M. (2021). Hair transplantation: basic overview. J Am Acad Dermatol..

[46] Rácz E., Gho C., Moorman P.W., Noordhoek Hegt V., Neumann H.A.M. (2013). Treatment of frontal fibrosing alopecia and lichen planopilaris: a systematic review. J Eur Acad Dermatol Venereol..

[47] Pindado-Ortega C., Saceda-Corralo D., Moreno-Arrones ÓM., Rodrigues-Barata A.R., Hermosa-Gelbard Á., Jaén-Olasolo P. (2021). Effectiveness of dutasteride in a large series of patients with frontal fibrosing alopecia in real clinical practice. J Am Acad Dermatol..

[48] Gómez-Zubiaur A., Valenzuela C., Andrés-Lencina J.J., Rodríguez-Villa A., Ricart J.M. (2022). Algorithm proposal for hair transplantation in fibrosing alopecia pattern distributidon. J Cosmet Dermatol..

