# Supplementary material for: Systematic review of efficacy of hair transplantation in patients with classic lichen planopilaris and frontal fibrosing alopecia: revisiting the current evidence
Source: An Bras Dermatol. 2026 Aug 12;101(5):501426. doi: 10.1016/j.abd.2026.501426 (PMC13495603; doi:10.1016/j.abd.2026.501426)
Supplement: Supplementary file 1 [file mmc1.docx]

**ABD-D-26-00185_Supplementary Material**

**Supplementary Table 1** Search strategy.

| **Search database** | **Search strategy** |
| --- | --- |
| PubMed | ((lichen planopilaris) OR ("frontal fibrosing alopecia") OR ("graham little piccardi lassueur")) AND (("hair transplantation") OR (transplantation)) |
| Web of Science | **(((TS=(lichen planopilaris)) OR TS=(Frontal fibrosing alopecia)) OR TS=(Graham-Little-Picardi-Lassueur)) AND TS=(hair transplant)** |
| SciELO | (liquen plano pilar) AND (trasplante) |
| Cochrane | (("lichen planopilaris"):ti,ab,kw OR (frontal fibrosing alopecia):ti,ab,kw OR (graham little piccardi lassueur):ti,ab,kw) AND (hair transplant):ti,ab,kw |

**Supplementary Table 2** 2011 Oxford center for evidence-based medicine levels of evidence.

| **Question** | **Step 1 (Level 1^a^)** | **Step 2 (Level 2^a^)** | **Step 3 (Level 3^a^)** | **Step 4 (Level 4^a^)** | **Step 5 (Level 5^a^)** |
| --- | --- | --- | --- | --- | --- |
| How common is the problem? | Local and current random sample surveys (or censuses) | Systematic review of surveys that allow matching to local circumstances^b^ | Local non-random sample^b^ | Case-series^b^ | n/a |
| Is this diagnostic or monitoring test accurate? (Diagnosis) | Systematic review of cross-sectional studies with consistently applied reference standard and blinding | Individual cross-sectional studies with consistently applied reference standard and blinding | Non-consecutive studies, or studies without consistently applied reference standards^b^ | Case-control studies, or poor or non-independent reference standard^b^ | Mechanism-based reasoning |
| What will happen if we do not add a therapy? (Prognosis) | Systematic review of inception cohort studies | Inception cohort studies | Cohort study or control arm of randomized trial^a^ | Case-series or case-control studies, or poor quality prognostic cohort study^b^ | n/a |
| Does this intervention help? (Treatment Benefits) | Systematic review of randomized trials or n-of-l trials | Randomized trial or observational study with dramatic effect | Non-randomized controlled cohort/follow-up study^b^ | Case-series, case-control studies, or historically controlled studies^b^ | Mechanism-based reasoning |
| What are the COMMON harms? (Treatment Harms) | Systematic review randomizes trials, systematic review of nested case-control studies, n-of-1 trial with the patient you are raising the question about, or observational study with dramatic effect | Individual randomized trial or (exceptionally) observational study with dramatic effect | Non-randomized controlled cohort/follow-up study (post-marketing surveillance) provided here are sufficient numbers to rule out a common harm. (For long-term harms the duration of follow-up must be sufficient)^b^ | Case-series, case-control, or historically controlled studies^b^ | Mechanism-based reasoning |
| What are the RARE harms? (Treatment Harms) | Systematic review of randomized rials or n-of-l trial | Randomized trial or (exceptionally) observational study with dramatic effect |  |  |  |
| Is this (early detection) test worthwhile? (Screening) | Systematic review of randomized rials | Randomized trial | Non-randomized controlled cohort/follow-up study^b^ | Case-series, case-control, or historically controlled studies^b^ | Mechanism-based reasoning |

^a^ Level may be graded down on the basis of study quality, imprecision, indirectness (study PICO does not match questions PICO), because of inconsistency between studies, or because the absolute effect size is very small; Level may be graded up if there is a large or very large effect size.

^b^As always, a systematic review is generally better than an individual study.

**Reference:** OCEBM Levels of Evidence Working Group. The Oxford Levels of Evidence 2. Oxford Centre for Evidence-Based Medicine [Internet]. Available in: https://www.cebm.ox.ac.uk/resources/levels-of-evidence/ocebm-levels-of-evidence
